# Proteomics as a Tool for the Study of Mitochondrial Proteome, Its Dysfunctionality and Pathological Consequences in Cardiovascular Diseases

**DOI:** 10.3390/ijms24054692

**Published:** 2023-02-28

**Authors:** Miroslava Stastna

**Affiliations:** Institute of Analytical Chemistry of the Czech Academy of Sciences, 602 00 Brno, Czech Republic; stastna@iach.cz

**Keywords:** proteomics, mitochondrial dysfunction, mitochondrial proteins, post-translational modifications, cardiovascular disease

## Abstract

The focus of this review is on the proteomic approaches applied to the study of the qualitative/quantitative changes in mitochondrial proteins that are related to impaired mitochondrial function and consequently different types of pathologies. Proteomic techniques developed in recent years have created a powerful tool for the characterization of both static and dynamic proteomes. They can detect protein–protein interactions and a broad repertoire of post-translation modifications that play pivotal roles in mitochondrial regulation, maintenance and proper function. Based on accumulated proteomic data, conclusions can be derived on how to proceed in disease prevention and treatment. In addition, this article will present an overview of the recently published proteomic papers that deal with the regulatory roles of post-translational modifications of mitochondrial proteins and specifically with cardiovascular diseases connected to mitochondrial dysfunction.

## 1. Introduction

The mitochondrial proteome consists of proteins that are encoded by both mitochondrial DNA and nuclear DNA. Mutations in the genomes that encode these proteins lead to protein alterations and protein deficiencies. The defected proteins that modulate mitochondrial behaviors and are associated with, e.g., mitochondrial dynamics, respiration and metabolism, mitophagy and mitochondrial protein import pathways, can cause mitochondrial dysfunction. Many diseases characterized by mitochondrial dysfunction have been described, e.g., cardiovascular disease [1,2], cancer [3], age-related metabolic and other disorders [4,5,6], neurological diseases [7] and insulin resistance/type 2 diabetes [8]. Apart from translated proteins that include alternatively spliced variants and isoforms [9], the mitochondrial proteome encompasses proteins that are regulated post-translationally, with single or multiple post-translational modifications (PTMs) of the same type or different PTM types, such as acetylation, succinylation and phosphorylation [10,11,12]. Importantly, PTMs are key mechanisms that not only expand the number of proteins and the proteome diversity, but they dynamically modulate mitochondrial function, activity, localization and interaction with other proteins. Reversible protein PTMs play regulatory roles after various extracellular/intracellular stimuli and during pathological changes [13,14,15].

Various mitochondrial activities and functions have been reported; however, for many, the underlying mechanisms at the protein level remain unclear. To be able to carry out the proper strategy for disease prevention and treatment, it is crucial to understand the cellular pathways that lead to the damage, as well as the protective processes, at the molecular level. Proteomics, as a powerful and efficient tool for identification/quantitation of the proteins, has been helping to clarify the involvement of mitochondrial proteins in various disorders [16,17,18]. In addition, the mitochondrial proteome is highly dynamic and it is subjected to dynamic changes in both quality and quantity. Thus, the progress in sample preparation, mass spectrometry instrumentation and bioinformatics enables the detection and quantification the levels of proteins with low experimental error and high accuracy. It can detect potential signaling pathways that can be targeted and it allows to determine the protein candidates for clinical intervention and treatment.

Cardiovascular diseases such as ischemia/reperfusion (I/R) injury, heart failure, cardiomyopathy or hypertension are associated with mitochondrial dysfunction that results from a defected respiratory chain, decreased adenosine-5′-triphosphate (ATP) synthesis, enhanced oxidative stress and changes in the mitochondrial structural integrity [19]. An impaired mitochondrial function is related to insufficient production of cellular energy and increased formation of reactive oxygen/nitrogen species (ROS/RNS) [19,20]. Although the pathways responding to mitochondrial energy deficits in high-energy tissues such as the heart have been recognized and various mechanisms have been shown to be effective in alleviation of cardiac injury, the specific mechanisms are not fully understood. Thus, the proteomic studies carried out recently have been able to shed the light on these mechanisms at protein levels, for example, during pre- or post-conditioning in I/R injury (short cycles of ischemia applied repetitively before or after a prolonged ischemic injury) [21,22].

The involvement of various mitochondrial proteins and mitochondrial dysfunction in different diseases has been reviewed [7,16,17,18,23,24,25], including cardiovascular diseases [19,26], as well as the roles of protein PTMs in health and their association with diseases [27,28,29,30,31,32,33,34,35,36]. The dysfunction of mitochondria associated with different aging-related diseases has been discussed as well [5,37].

In this review, mitochondria and their metabolic pathways are overviewed briefly, followed by a discussion on mitochondrial proteins (mitoproteome). Mass spectrometry (MS)-based proteomic approaches for mitochondrial protein identification/quantitation are described and databases that can support the research on mitochondrial proteins are included. Furthermore, examples of proteomic studies performed over the last five years on mammals are discussed with a focus on mitochondrial dysfunction, involvement of mitochondrial proteins, regulation roles of PTMs and cardiovascular diseases.

## 2. Mitochondria

The mitochondrial structure of most eukaryotic cells contains the following compartments: the outer mitochondrial membrane (OMM), the intermembrane space (IMS) and the inner mitochondrial membrane (IMM), forming the cristae and the matrix. The number and size of mitochondria are dependent on cell type, tissue and organism; however, the size is usually in range of 0.5–1 µm. Muscle cells that have to fulfill large energy requirements during movement contain a large number of mitochondria. In addition, the heart muscle cells are rich in mitochondria, with a content of about 5000 per cell, as the heart muscle has high energy demands to pump blood via the circulatory system. The major task of mitochondria is production of ATP and providing energy to cells; however, “mitochondria are intimately embedded in the signaling cascades and programs that operate within the cells” [38]. For example, the mitochondrial function is modulated by cellular demand and the changes in cell metabolism, development and death are responded to by the mitochondrial function, localization and biogenesis. The mitophagy pathway is an essential quality control mechanism for clearance of damaged mitochondria [39]. In addition, mitochondria synthesize many biomolecules and they are involved in, e.g., cell signaling (via release of ROS/RNS, calcium, etc.), membrane potential regulation, apoptosis-programmed cell death, regulation of immunity and other processes [5,38,40,41]. Fusion and fission processes are important for mitochondria function and have a role in apoptosis [42].

Healthy mitochondria are essential for proper organ functionality. Mitochondria under stress conditions trigger signaling pathways to activate the cell response, and various mediators of mitochondrial communication are released, such as metabolites of the mitochondrial metabolism system [43,44]. However, defective mitochondria have reduced abilities to communicate within the cell which can cause energy and cellular mechanism changes, eventually leading to cell death. Defects in the energy production system, especially in organs with high demands on energy such as the heart, lead to mitochondrial disorders that are related to cardiac diseases. Thus, many cardiac diseases are characterized by mitochondrial dysfunction. Mitochondria produce 95% of cellular ATP, which supports the life-long cycles of heart beats [1,45]. Mitochondria dysfunction in cardiac diseases is connected to a bioenergetic imbalance in ATP synthesis and ATP hydrolysis, increased production of ROS, a defective electron transport chain (ETC) system, altered ion homeostasis (Ca^2+^) and release of proapoptotic proteins [23,42,46]. The deficiency in heart energy caused by mitochondrial dysfunction contributes to cardiomyopathies and heart failure (HF) [42]. In addition, early structural changes in mitochondria can be observed with depletion of cytoplasmic glycogen particles and swelling of mitochondria in cardiomyocytes during ischemia [47].

### 2.1. Mitochondrial Energy Production

To provide energy to cells in the form of ATP, the cardiac mitochondria depend on metabolic pathways, specifically on three major pathways (cellular respiration), i.e., fatty acid β-oxidation (FAO), the tricarboxylic acid cycle (TCA cycle, Krebs cycle and citric acid cycle) and the oxidative phosphorylation (OXPHOS) system. In FAO, acetyl-coenzyme A (acetyl-CoA) is produced together with the reduced form of nicotinamide adenine dinucleotide (NADH) or the reduced form of flavin adenine dinucleotide (FADH_2_). Prior to FAO, long-chain acyl-CoA fatty acids from cytosol are activated by acyl-CoA synthetases and are imported inside mitochondria by the carnitine cycle [48]. The carnitine cycle consists of two carnitine palmitoyl transferases (CPT1 and CPT2) and carnitine acylcarnitine translocase, CACT (SLC25A20). CPT1 is an integral enzyme of the OMM which catalyzes conversion of acyl-CoA to acylcarnitine and CACT mediates the transport of acylcarnitine to CPT2, a peripheral protein of IMM, which converts acylcarnitine back to acyl-CoA. A long-chain acyl-CoA inside mitochondria enters four steps of the FAO cycle, in which acyl-CoA is degraded, its molecule is sequentially shortened and it finally results in the production of acetyl-CoA, as well as the reduced products FADH_2_ and NADH that subsequently enter the TCA cycle. Acyl-CoA degradation is catalyzed by different acyl-CoA chain length-specific enzymes (acyl-CoA dehydrogenases; AD): very long chain (VLCAD), medium-chain (MCAD) and short-chain (SCAD) acyl-CoA dehydrogenases in humans [21,48]. In the TCA cycle, inside the mitochondrion matrix, acetyl-CoA is further oxidized and NADH, FADH2 and guanosine-5′-triphosphate (GTP) are produced with the release of CO_2_. NADH and FADH_2_, as donors of electrons, are together with O_2_ used in the OXPHOS system, in which electrons are transferred from electron donors to electron acceptors during a set of redox reactions. The OXPHOS system consists of five protein complexes (complexes I–IV and FoF1 ATP synthase complex) that are bound to IMM. Protein complexes I–IV (ETC) are respiratory chain complexes. They are NADH dehydrogenase (NADH-ubiquinone oxidoreductase; complex I), succinate dehydrogenase (complex II), cytochrome c oxidoreductase (cytochrome b and c1; complex III) and cytochrome c oxidase (complex IV). Electrons enter complex I and are transferred to complex IV, in which O_2_ is the final electron acceptor and H_2_O is produced (reduction of molecular oxygen to water). The energy from the redox reactions is used predominantly for transporting the protons from the mitochondrial matrix to IMS and an electrochemical proton gradient is created across the IMM which is used by the FoF1 ATP synthase complex to synthesize ATP through phosphorylation of adenosine diphosphate (ADP) [21,49]. The ATP synthase complex FoF1 is not a respiratory chain complex as it is not involved in electron transfer. It is an independent complex in which ATP synthase consists of two parts: an oligomycin sensitive proton channel (Fo) embedded in the IMM and a catalytic subunit F1 directed into the matrix.

When insufficient molecular oxygen delivery occurs, for example, during cardiac ischemia, the reducing components of ETC that are normally re-oxidized fail to follow this process. As a result, the electron flow in the ETC decreases and this has a negative effect on both the FAO pathway and the TCA cycle progress. ATP production by the OXPHOS system is compromised and this phenomenon forces the cells to switch to anaerobic glycolysis, the alternative way to produce ATP energy.

The functions of mitochondrial membrane protein complexes and their assembly into macromolecular structures (supercomplexes) [50] have been reviewed [7,51,52], and their structures have been analyzed and visualized by electron cryo-tomography [53].

### 2.2. Mitochondrial Proteome

A mitochondrion possesses its own maternally inherited genome (mitochondrial DNA; mtDNA) and contains mitochondrial proteins that are encoded by mtDNA; however, during evolution, the number of protein coding genes in mtDNA was substantially reduced in eukaryotic cells. In humans, 13 proteins are encoded by mtDNA. These hydrophobic proteins are core subunits of OXPHOS complexes that are inserted into IMM during their synthesis, either peripherally or integrally. They include seven subunits of complex I (chains 1–6 and chain 4 L of NADH-ubiquinone oxidoreductase (ND1-ND6 and ND4L)), one subunit of complex III (cytochrome b), three subunits of complex IV (subunits 1–3 of cytochrome c oxidase (COX1-COX3)) and two subunits of FoF1 ATP synthase complex (ATP synthase subunit a (ATP6) and ATP synthase protein 8 (ATP8)) [54]. However, over 1100 mitochondrial proteins (98% of all mitochondrial proteins that have been identified to date) are encoded by nuclear genes, synthesized in cytosol and subsequently imported into mitochondria by a set of protein translocases and associated proteins. Two experimental explanations have been given in order to explain why these proteins are expressed in mitochondria, even though their expression requires many nuclear encoded proteins to be imported from cytosol to mitochondria: (i) the high hydrophobicity of the proteins prevents their efficient import from cytosol to IMM [55] and/or (ii) regulation of the levels of de novo protein synthesis to amounts that can be assembled (regulation of gene expression by coupling synthesis and assembly) [54,56]. The proteins of mitochondria are embedded or associated with both mitochondrial membranes, and the soluble proteins reside in the matrix and IMS. The OXPHOS complexes include about 80 proteins, and they require additional assembly proteins and factors for their synthesis [57]. For example, in complex I, 14 central subunits that have bioenergetic activities are enveloped by 30 accessory protein subunits [52]. However, only two accessory subunits have known structures [52,58]. The list of the subunits of complex I can be found in [52] for different species. Mammalian complex III includes eleven subunits with three major subunits that catalyze electron transfer (cytochrome b, cytochrome c_1_ and the Rieske iron-sulfur protein). The other subunits stabilize and regulate complex III and contribute to efficient electron transfer [59]. About 20 protein components that participate in the human mitochondrial FAO pathway with their enzymes and transporters are listed in [48], i.e., five proteins participating in carnitine shuttle, eleven proteins of the FAO pathway and four auxiliary enzymes.

Mitochondrial dysfunction-mediated diseases are in many instances caused by mutations in nuclear and/or mitochondrial genomes encoding the proteins that were part of the complexes during their assemblies or the proteins that participated in complex expression [38]. Mitochondrial dysfunction caused by unusually expressed proteins participating in mitochondrial dynamics and activities, such as bioenergetics, import machinery, autophagy (mitophagy), ion channels, quality control and others, can consequently result in different pathologies [25]. For example, mutation in NADH dehydrogenase (ubiquinone) iron-sulfur protein 6 (NDUFS6; highly conserved subunit of complex I that participates in complex I assembly) causes mitochondrial complex I deficiency [60], a disease that leads to clinical disorders such as cardiomyopathy, contractile function and HF [23,61,62]. It was observed that deletion of cytochrome b-c1 complex subunit 1 (UQCRC1; a subunit of complex III encoded by nuclear DNA) caused mitochondrial dysfunction, whilst overexpression of UQCRC1 enhanced complex III activity and had a protective function in cardiomyocytes [63].

Efforts have been made in the past to compile mitochondrial protein datasets, including the proteins that make up complex I for different species [52], proteins that participate in the FAO pathway [48], mitochondrial proteins extracted from heart and liver tissues [64] and mitochondrial proteome from different rat tissues of the liver, kidney, brain and heart [65]. These datasets, however, could suffer from several problems such as false positive rates when MS-based experiments were performed, identification of co-purified mitochondrial contaminants and difficulties connected to detection of low abundance proteins. Over the years, an extensive catalogue of human heart mitochondrial proteins has been published [66], and a mitochondrial protein compendium, termed MitoCarta, has been generated [67]. Mito Carta1.0 (2008) and MitoCarta2.0 (2015) were updated in MitoCarta3.0 (2020) [68], containing 1136 human and 1140 mouse genes encoding proteins, mitochondrial localization, manually curated annotations of sub-mitochondrial localizations and 149 pathway annotations. Additionally, the mitochondrial proteome database MitoP2 was established in 2006, which now includes information on mitochondrial proteins, their molecular functions and the related pathologies for yeast, humans and mice [69]. MitoProteome is another mitochondrial protein sequence database and annotation system [70] that includes information manually annotated and extracted from external databases (e.g., LocusLink, Ensembl, disease information from OMIM (Online Mendelian Inheritance in Man), protein–protein interactions from MINT (Molecular INTeraction database) and the DIP (Database of Interacting Proteins), protein fingerprints from PRINTS, structural data from the PDB (Protein Data Bank), mutation data from the PMD (Protein Mutant Database) and blast homology data from NCBI and Swiss-Prot). Furthermore, MitoMiner is an integrated database introduced in 2009 [71] that was updated to MitoMiner v4.0 in 2018 [72] and provides experimental subcellular localization protein datasets with tissue expression, predictions of targeting sequences, gene annotations and connection to diseases. The human mitochondrial high-confidence proteome dataset (MitoCoP) was published in 2021 and includes > 1100 mitochondrial proteins [73]. Although not specifically targeted to mitochondrial proteins, an atlas of tissue-specific protein phosphorylation has been assembled [74], with over 6000 phosphoproteins harboring about 36,000 phosphorylation sites. This can be a good source for mitochondrial phosphoprotein research as well. Examples of articles that include lists of mitochondrial proteins as well as mitochondrial protein databases available online are summarized in Table 1 with additional information. Although other mitochondrial gene/protein databases have been introduced, e.g., GOBASE, MITOP or HMPD, they are not functioning any longer, and thus are not included in Table 1.

Currently, biological databases (not purely mitochondrial) that could further support research on mitochondrial proteins and could serve as a public data repository are available. For example, these include the PRIDE (PRoteomics IDEntifications; public repository for protein expression data determined by mass spectrometry), KEGG (Kyoto Encyclopedia of Genes and Genomes), STRING (Search tool for the retrieval of interacting genes; known and predicted protein–protein interactions; https://string-db.org (accessed on 10 January 2023)), UniProt (Universal protein resource), Pfam (protein families database), Ensembl (a genome browser for vertebrate genomes), GO (gene ontology consortium), CDD (conserved domain database for proteins), InterPro (protein sequence analysis and classification), RefSeq (Reference Sequence database), IPA (Ingenuity Pathway Analysis; the curated database that enables to predict protein interactions and pathways based on previous knowledge and reported relationships from the literature) and PhosphoSitePlus (PSP; the knowledgebase dedicated to PTMs such as phosphorylation, acetylation, ubiquitylation and methylation). Many of these databases can be found in Database Commons—a curated catalog of worldwide biological databases (https://ngdc.cncb.ac.cn/databasecommons/ (accessed on 10 January 2023)). Other sources for human mitochondrial proteins are the Human Proteome Map (http://humanproteomemap.org/ (accessed on 10 January 2023)) and the Human Proteome Atlas (http://www.proteinatlas.org/ (accessed on 10 January 2023)).

## 3. Identification of Mitochondrial Proteins

Various strategies have been developed to identify the mitochondrial proteome. It is highly dynamic, as the levels of protein expression and localization, as well as modifications by multiple PTMs, reflect the responses to various stimuli and pathological conditions. Due to the broad dynamic range, proteome sub-fractionation is often performed in order to simplify the complex mixtures. As many proteins are present in very low abundancies, different methods have been applied for protein enrichment with subsequent identification.

### 3.1. Mitochondria Isolation and Purification Quality

Usually, after cell/tissue homogenization, differential centrifugation is applied to isolate mitochondria from the rest of the cellular components, the procedure of which depends on the cell/tissue types. First, centrifugation is performed at low speed to pellet the intact cells, tissue debris and nuclei (600–800× *g*) followed by high-speed centrifugation to pellet and concentrate the mitochondria (10,000–26,000× *g*). Furthermore, centrifugation in density gradient (sucrose gradient or Percoll gradient; 30,000–100,000× *g*) is used on crude mitochondria pellets or affinity purification is included to obtain purer mitochondria [24,75,76]. The purity of isolated mitochondria can be validated by the presence of markers for mitochondria (e.g., cytochrome c oxidase subunit 4 (COX4), voltage-dependent anion-selective channel protein 1 (VDAC1), NADH dehydrogenase (ubiquinone) 1 beta subcomplex subunit 11 (NDUFB11), etc.) and the absence of non-mitochondrial proteins using, e.g., Western blotting. Contamination from other cellular components is always an issue; however, the highly sensitive liquid chromatography-tandem mass spectrometry (LC-MS/MS) instrumentation and bioinformatic tools that are used nowadays enable sorting and identifying mitochondrial proteins from the whole cell proteome [12,13]. Still, the quantitation of the absolute abundance of mitochondrial protein that has multiple cell localization can be difficult since whole cell proteomes contain both forms of the protein (mitochondrial as well as non-mitochondrial form) [77]. Thus, in many instances, and especially for proteins with questionable localization, the purity of the mitochondrial preparation can be crucial to avoid misidentification of the proteins, and proteomic studies of isolated mitochondria and their sub-compartments have been carried out [66,77]. Recently, an extensive approach has been used that was able to identify high-confidence human mitochondrial and mitochondria-associated proteomes with experimental validation, including proteins with multiple cell localization [73]. This approach combined subtractive proteomics (mitochondrial preparations of different purity), spatial (subcellular) proteomics, mitochondria-specific importomics (imported via TOMM40) and literature/database curation of HEK293T, HeLa, Huh7 and U2OS human cell lines [73].

### 3.2. Mass Spectrometry-Based Proteomics of Mitochondrial Proteins

In recent years, numerous proteomic techniques have been developed for identification/quantitation of proteins of various origins. Proteomic methods applied for separation, identification and quantitation of mitochondrial proteins are traditionally two-dimensional gel electrophoresis (2-DE) followed by mass spectrometry (MS). Despite the limitations related to 2-DE, such as the detectability of low abundance proteins and difficulty of dissolving hydrophobic membrane proteins, it is useful for the detection of protein PTMs, where the shift in the isoelectric points, as well as the protein distribution, can be visualized on the gels. In this instance, the isolation/enrichment of mitochondria from cell lysates/homogenates is crucial and can be followed, e.g., by two-dimensional fluorescence difference gel electrophoresis (DIGE) [78] and, if needed, by MS. In DIGE, proteins from different samples are covalently labeled, each sample with a different fluorescent CyDye label, then the samples are mixed, the proteins are separated and their different positions are visualized on the same gel with high sensitivity.

To enrich/purify the mitochondrial proteins/peptides, immunoaffinity methods are applied, for example, enrichment of acetylated and malonylated tryptic peptides from heart tissue homogenate [13] and enrichment of mitochondrial DRP1 protein with subsequent enrichment of tryptic DRP1 peptides using acetyllysine antibody [15].

MS approaches for protein/peptide identification and quantitation include tandem instruments, such as matrix-assisted laser desorption/ionization-time of flight (MALDI-TOF/TOF) for peptide mass fingerprinting or LC-MS/MS with further peptide fragmentation in order to obtain amino acid sequences. LC-MS/MS strategies for protein quantitation are label-free techniques [79], chemical labeling techniques, e.g., isobaric tags for relative and absolute quantification (iTRAQ) [80], and metabolic labeling methods, such as stable isotope labeling by amino acids in cell culture (SILAC; ^13^C_6_, ^15^N_4_ arginine, ^13^C_6_ lysine or ^13^C_9_ tyrosine) [81] or labeling the proteins using non-canonical amino acids (azidohomoalanine (AHA) and homopropargylglycine (HPG)) [82]. In label-free MS-based methods, two approaches are applied: counting the number of peptides/spectra assigned to a particular protein (spectral counting method) or measurement of ion abundances (peak intensities) of the peptides (ion intensity method). The quantitation of the differential protein expression is derived from a comparison of the spectral counts of the same protein in compared samples or from a comparison of the peak intensities of the same peptide in compared experiments. In the non-canonical labeling method, AHA or HPG (azide or alkyne amino acid analogs of methionine) are incorporated into proteins with subsequent copper-catalyzed azide-alkyne ligation (Click-iT reaction) that enables affinity enrichment of AHA/HPG-labeled proteins/peptides. Both SILAC and AHA/HPG can be used for labeling and identification of newly synthesized proteins in vitro and in vivo and, compared to the iTRAQ method (in which the samples are processed in parallel), there is a minimal variation between samples as they are labeled at the cell/organism level. In SILAC, there are several cell divisions needed to fully incorporate the label, whilst in the AHA/HPG labeling method, the label incorporation is fast and allows for detection of changes in protein abundances soon after their synthesis (dynamic proteomics). In addition, AHA/HPG labeling protocols include a protein/peptide enrichment step which is important for identification of low abundance, newly synthesized proteins. More details about the principles of label-free labeling and metabolic labeling by SILAC and AHA/HPG can be found in [79,81,82,83,84].

In discovery-driven proteomics, nano-liquid chromatography coupled to a tandem mass spectrometer via electrospray ionization (nLC-MS/MS) is applied. Usually, data-dependent acquisition (DDA) mode is implemented in which a survey MS scan is first performed on the sample of enzymatically digested peptides and then a fixed number of peptide precursor ions is selected and analyzed by MS/MS. The resulting spectra of MS/MS fragment product ions are searched and matched to database protein sequences.

The acquisition strategy, called “sequential window acquisition of all theoretical fragment-ion spectra” (SWATH) [85,86], can be used to target and to identify/quantify mitochondrial proteins, and it is a tool that allows for unambiguous assignments of PTMs as well as protein isoforms. In a single run, the method performs consecutive survey scans and produces fragment ion spectra of all precursor ions within the series of isolated “swaths” (windows) with pre-determined retention times and m/z ratios. Then, it compiles them into complex maps of fragment ions with fragment ion m/z ratios, retention times and intensities. The method uses data-independent acquisition (DIA), which means that to identify and quantify the peptides from the maps of fragment ions, a targeted data extraction method is needed, in which the acquired experimental data are compared to the information stored in MS spectral libraries for various cells and species [85].

Another targeted MS-based method that can selectively analyze specific proteins, protein isoforms and PTMs or can be used as an antibody-free validation tool is multiple (selected) reaction monitoring (MRM and SRM) [87]. MRM is a sensitive and accurate method and can be used for detection and quantitation of targeted peptides that represent a specific protein in a complex matrix. It requires a priori information on the proteins/peptides of interest to design the assay for each target protein in which the corresponding pairs of parent–product ions (transitions) are selected for various peptides. MRM has been used for, e.g., validation of mitochondrial proteins in dilated cardiomyopathy [88] and for quantifying cardiac mitochondrial protein phosphorylation [89]. More information on mitochondrial proteomics can be found in review articles [17,24,31].

## 4. Regulatory Roles of Post-translational Modifications

Regulation of protein expression and PTMs are the key events, for example, in the development and maturation of the heart, in neonatal cardiomyocyte proliferation and in maturation and differentiation. It was documented that “typical” phosphoproteins are expressed across tissues, yet they display tissue-unique phosphorylation sites from various kinases that modulate protein function in order to comply with the specific needs of a particular tissue type [74]. PTMs are essential in temporary regulation of mitochondrial protein functions and interactions as well as enzyme activities. In the following sections, examples of two mitochondrial protein PTMs are overviewed, acylation and phosphorylation, which are related to mitochondrial dysfunction and various heart diseases. More details are included on proteomic studies that have been published in the last five years.

### 4.1. Acylation

One of the PTMs that targets mitochondrial proteins is acylation. Generally, it is a reversible covalent addition of an acyl group from acyl-CoA to a lysine residue of the protein molecule. Experiments have shown that the mitochondrial proteome is highly acylated and the addition of various acyl groups to the proteins results in various PTMs, i.e., the addition of an acetyl group from acetyl-CoA results in acetylation, the addition of a succinyl group from succinyl-CoA results in succinylation, the addition of a malonyl group from malonyl-CoA results in malonylation and the addition of a glutaryl group from glutaryl-CoA results in glutarylation. Although these PTMs are under the same umbrella of structurally similar acylation, they have various effects on the protein structure, function and interaction with other proteins and are the reasons for different pathological consequences. These PTMs modify different proteins in different biological pathways [43,44]. Protein acetylation links metabolism and cell signaling [43] and it is driven by acetyl-CoA from the FAO pathway [44]. The stress-response sirtuin family of proteins are known to act as deacylases, e.g., SIRT3 (NAD-dependent protein deacetylase sirtuin-3) acts as a deacetylase and SIRT5 (NAD-dependent protein deacylase sirtuin-5) has enzymatic activities as deacetylase, desuccinylase and demalonylase. For example, non-proteomic studies have shown that mitochondrial SIRT3-deficient mice exhibited hyperacetylation of mitochondrial proteins (but not SIRT4- or SIRT5-deficient mice) [90], showing SIRT3 as a mitochondrial deacetylase and the protein that regulates reversible protein acetylation in mitochondrion. Using MS-based proteomics, 84 mitochondrial proteins have been shown to be increasingly acetylated in SIRT3-deficient mice (six enzymes of FAO, fifty subunits of ETC and three enzymes of TCA) [91], causing impairment of mitochondrial and contractile functions in the myocardium, mainly due to up-regulated acetylation of metabolic proteins and depletion of myocardial energy [91]. Another MS-based study has reported that SIRT5 deacylated proteins that are involved in the cellular oxidative mechanism in order to regulate mitochondrial energy production [92], and SIRT5 was required for mouse survival in response to cardiac pressure overload induced by transverse aortic constriction (TAC) [92]. By profiling the levels of acyl-CoA in various mouse tissues using MS-based proteomics, different acyl-CoA profiles have been identified with succinyl-CoA as the most abundant in the heart [9]. Affinity enrichment, proteomics and MS analysis were applied [93] to identify succinylated lysine residues in the proteins of mitochondria that were isolated from SIRT5-deleted mouse hearts (289 unique succinylated lysine residues in 46 proteins identified) and WT mouse hearts (44 succinylated lysine residues in 5 proteins detected). In addition, another study has reported a shift in cardiac energy metabolism from glycolysis to the FAO pathway during heart maturation, to which the changes in acetylation and succinylation of the metabolic enzymes contributed [94]. Succinylation has been hypothesized to regulate energy metabolism during atrial fibrillation [95].

The signaling pathways activated in the heart as a result of mitochondrial energy dysfunction have been investigated in mice with depleted mitochondrial phosphate carrier protein, SLC25A3 [13]. This mice model is characterized by defective ATP synthesis in mitochondria, eventually resulting in development of cardiomyopathy. In [13], remodeling of acylome was observed in SLC25A3-depleted hearts compared to hearts with SLC25A3 (control) using Western blotting. Significantly up-regulated protein acetylation and malonylation was identified, whilst protein succinylation and glutarylation remained unaltered. Specifically, mouse cardiac proteins from heart tissue were extracted and the proteins were first digested by Lys-C for 4 h followed by overnight trypsin digestion. The peptides were subjected to immunoaffinity purification, and enriched acetylated and malonylated peptides were separated by nLC-MS/MS (Dionex Ultimate 3000 RSLC coupled to Orbitrap Fusion Tribrid MS (Thermo Fisher Scientific, Waltham, MA, USA)). MS identified 94 and 153 proteins that were hyperacetylated and hypoacetylated, respectively, in the SLC25A3-deleted group compared to the control. In addition, 39 differentially acetylated proteins in the SLC25A3-deleted group exhibited dual acetylation status at lysine sites (i.e., increased and reduced acetylation) within the same protein. Furthermore, 68 proteins were differentially malonylated in the SLC25A3-deleted group and all of them were hypermalonylated compared to the control. Interestingly, 22 proteins showed increased acetylation, reduced acetylation and increased malonylation at lysine sites within the same protein. Using the Mitocarta3.0 database, 91% of hyperacetylated proteins and 73% of hypermalonylated proteins were annotated as mitochondrial proteins. For example, the enzyme isocitrate dehydrogenase 2 (IDH2) was both highly acetylated (five sites) and highly malonylated (nine sites) in response to SLC25A3 deletion. Three lysine sites dually modified by both acetylation and malonylation in IDH2 (K180, K263 and K48) in response to SLC25A3 deletion were further examined in terms of enzyme function dependency on the modification site. Creating the acylation mutant constructs of IDH2 and WT IDH2 that were transiently re-expressed into HEK293 IDH2 knockout cells, it was found that malonylation and acetylation at K263 had no impact on IDH2 function in cell mitochondria as well as malonylation at lysine site K48; however, acetylation at K48 resulted in elevated activity of IDH2 compared to WT re-expressing mitochondria. For lysine site K180, a mitochondria re-expressing malonylated K180 mutant and an acetylated K180 mutant exhibited decreased IDH2 activity and enhanced IDH2 activity, respectively; thus, acetylation and malonylation at K 180 had opposite effects on the IDH2 function. To summarize, acetylation of IDH2 increased enzyme activity and malonylation of IDH2 decreased enzyme activity. The authors concluded that cumulative effect of SLC25A3 deletion-induced specific hyperacetylation and hypermalonylation of IDH2 enhanced enzyme activity [13]. Furthermore, in the same study, the quantitation of the components of the pathways that control protein acetylation/deacetylation (acetyl-CoA, GCN5L1 and SIRT3) and protein malonylation/demalonylation (malonyl-CoA and SIRT5) were investigated using targeted LC-MS/MS [13]. As a result, SLC25A3 deletion did not alter acetyl-CoA (the substrate for acetyl groups) levels; however, it decreased the expression of GCN5L1 (mitochondrial acetyltransferase) and decreased the levels of SIRT3 (mitochondrial deacetylase). These data implied that in SLC25A3-deleted hearts, hyperacetylation occurred because of reduced SIRT3 expression. SLC25A3-deleted hearts showed elevated malonyl-CoA (the substrate of malonyl group) and unaltered SIRT5 (mitochondrial demalonylase) levels; however, SIRT5 was hyperacetylated at lysine site K203. These data suggested that SIRT5 acetylation at K203 reduced SIRT5′s function as a deacylase. The results obtained from subsequent experiments, in which re-expressed SIRT5 acetylated K203 mutants and a WT SIRT5 mutant were prepared, confirmed the conclusions derived from the MS data. In summary, this work showed that defective mitochondrial ATP synthesis induced by cardiomyocyte-specific SLC25A3 deletion increased protein acetylation, currently known to be associated with dysfunction of mitochondrial energy in other models of cardiac diseases, and concomitantly increased mitochondrial protein malonylation. Thus, a novel cross-talk between two modifications was observed, in which energy dysfunction-induced acetylation of deacylase SIRT5 inhibited its activity, implying that acetylation can control the malonylome [13].

It has been reported that lysine acylation of mitochondrial proteins contributed to heart failure by impairment of the respiratory function and the oxidative metabolism [92,96,97]. However, there has been growing evidence that despite extreme lysine acylation in heart mitochondria, the effect on bioenergetics is minimal [98]. In addition, mice models with SIRT3-deletion or SIRT3-manipulation showed that the associated dysfunction appeared to be specific to the type of metabolic stress [99,100], for example, it failed to affect insulin secretion or β cell metabolism in the absence of overnutrition [100]. Recently, the study of mitochondrial bioenergetics and the role of mitochondrial hyperacetylation in a TAC model of cardiac pressure overload has been examined [11]. In [11], a genetic dual knockout (DKO) mouse model of extreme mitochondrial acetylation in cardiac muscles was created, with deficiencies in two enzymes that oppose lysine acetylation, SIRT3 and carnitine O-acetyltransferase (CrAT) enzyme. In addition, DKO mice were subjected to TAC in order to induce cardiac pressure overload and evaluate the susceptibility of DKO mice to HF. In addition, a second experimental mice model of heart failure using TAC with a small apical myocardial infarction (TAC-MI) was created and compared to the genetic model. Quantitative proteomics was used to access lysine acetylation in the mitochondrial proteome and the effect of acetylation on mitochondrial bioenergetics was evaluated, as well as the possibility of HF in DKO mice. The mice cardiac tissues were lysed and the proteins were digested using trypsin and LysC. The resulting peptides were labeled by tandem mass tag TMT reagents (peptide labeling with isobaric tags; Thermo Fisher) that enabled direct comparison of the relative abundances of acetylated peptides between the samples. Compared samples were mixed together and subjected to nLC-MS/MS (EASY-nLC 1200 coupled to Q Exactive Plus Hybrid Quadrupole Orbitrap MS; Thermo Fisher). The obtained data showed that in DKO mice, the quantity of mitochondrial acetylated lysine residues was additively enhanced by SIRT3 and CrAT depletion (compared to mice with only single deficiency of SIRT3; SIRT3 KO mouse model). By a comparison of the experimental (TAC-MI) model with a genetic DKO mice model without TAC, it was found that 86% of mitochondrial acetylated residues identified in (TAC-MI) mice were detected in the DKO model as well. A total of 85% of mitochondrial acetylated residues in TAC-MI mice were up-regulated compared to the surgical sham control. Furthermore, 88% of 378 mitochondrial acetyl-peptides upregulated in (TAC-MI) versus sham (fold change FC ≥ 1.5) were found in 614 acetyl-peptides elevated in DKO mice model versus control (FC ≥ 1.5). A quantitative comparison showed that 94% of quantified mitochondrial acetylated lysine sites were more abundant and robust in the DKO model compared to TAC-MI mice with an average FC of 2.4. Mitochondrial diagnostics using a bioenergetics assay platform, including a creatine kinase bioenergetic clamp, was performed to evaluate the function outcomes of mitochondrial acetylation and potential disruption of the oxidative metabolism. Despite extreme lysine hyperacetylation in DKO mice, there was a minimum impact on mitochondrial bioenergetics (i.e., oxygen consumption, mitochondrial membrane potential, NAD(P)^+^/NAD(P)H redox state and ROS emission) compared to the control. Moreover, experiments on DKO animals subjected to TAC surgery showed that dual deletion of SIRT3 and CrAT did not worsen cardiac dysfunction and disease progression when coupled with pressure overload induced by TAC. In summary, the acetylome in the DKO model not only recapitulated but highly increased the magnitude of hyperacetylation identified in a pressure overload model of HF (TAC-MI). In DKO mice with extreme acetylation, minimal evidence of oxidative dysfunction was found compared to other conditions examined. Using TAC-induced pressure overload, DKO hearts were not more susceptible to dysfunction. Thus, based on the experiments in this study, acetylation of the cardiac mitochondrial proteins did not contribute to heart failure and although hyperacetylation of the mitochondrial proteome in the heart can lead to functional decline, it was not sufficient to cause pathological remodeling during pressure overload using TAC [11].

In another work, the effect of SIRT3 downregulation on the mitochondrial acetylome in vivo induced by doxorubicin treatment was examined using MS [14]. Doxorubicin is a chemotherapeutic given to cancer patients; however, its usage is restricted as it is connected to development of dilated cardiomyopathy (DC). In DC, the left ventricular chamber of the heart is enlarged with a thinner ventricular wall caused by cardiomyocyte death, and it leads to systolic and diastolic dysfunction. Two types of transgenic mice were created [14], each with different SIRT3 isoforms, either the shorter M3-SIRT3 isoform (truncated SIRT3; deacetylase function but poorly localized in mitochondria) or the M1-SIRT3 isoform (full length; main mitochondrial deacetylase localized in the mitochondria). Ten-week-old transgenic mice and nontransgenic mice were administered doxorubicin for 4 weeks and compared against a non-transgenic saline-treated control group. Mitochondrial fractions from heart tissues were isolated/enriched using an isolation kit (MITOISO1; Sigma-Aldrich) and the proteins were digested by the trypsin Lys-C mix. The resulting peptides were immunoprecipitated by an acetyl-lysine antibody bound to agarose beads and acetylated peptides were isolated. An analysis of acetylated peptides was performed by use of an Orbitrap Q Exactive HF-X MS (Thermo Fisher Scientific, Waltham, MA, USA) with DDA mode and label-free quantitation. In their previous work, the authors reported that increased expression of M1-SIRT3 reduced doxorubicin-induced ROS production in mitochondria and improved mitochondrial respiration in rat H9c2 cardiomyocytes [101]. In [14], their work was extended to experiments in vivo with the hypothesis that SIRT3 expression could reduce doxorubicin-induced DC by the decrease in the acetylation of mitochondrial proteins. The experiments confirmed that doxorubicin highly remodeled the cardiac mitochondrial acetylome in non-transgenic doxorubicin-treated mice compared to the saline control group. Using echocardiography, it was observed that M1-SIRT3 transgenic mice exhibited a resistance to cardiac remodeling and dysfunction induced by doxorubicin treatment, whilst M3-SIRT3 transgenic mice were only partially resistant to the doxorubicin-induced changes. Proteins detected with altered acetylation sites in doxorubicin-treated hearts (37 unique acetylation sites were altered) were associated with oxidative and metabolic stress resistance. For example, superoxide dismutase 2 (SOD2; oxidative stress resistance protein) and IDH2 (regulator of oxidative stress in the heart) exhibited altered acetylation as a result of doxorubicin treatment. In the SOD2 protein, six acetylation sites in six peptides were identified, with K122 showing down-regulated acetylation and K130 showing up-regulated acetylation in doxorubicin-treated hearts. In addition, the levels of SOD2 were decreased in non-transgenic mice treated with doxorubicin, whilst M3-SIRT3 and M1-SIRT3 mice showed unchanged levels of SOD2. This implied that SIRT3 can modulate the changes induced by doxorubicin during oxidative stress via regulation of acetylation of SOD2 at K122 and K130 sites and by regulation of SOD2 levels. In conclusion, doxorubicin decreased SIRT3 expression and had an impact on mitochondrial acetylome in the heart. The enhanced expression of M1-SIRT3 in vivo prevented cardiac dysfunction induced by doxorubicin treatment. Thus, mitochondrial SIRT3 can reduce doxorubicin-induced DC by modulation of protein acetylation and oxidative stress [14].

The function and acetylation of a mitochondrial fission regulator, dynamin-related protein 1 (DRP1), have been investigated in a model of lipid overload-induced cardiomyocyte death and myocardium dysfunction [15]. Here, mice and monkey animal models as well as cell culture models were examined. Mice were fed with a high-fat diet (HFD) for 18 weeks and monkeys were fed with a diet of high-fat and high-cholesterol (HFHC) for 2.5 years. The groups were compared to control diet (CD)-fed animals. To determine DRP1 acetylation in heart samples, whole heart tissues were lysed, DRP1 was isolated using anti-DRP1 antibody beads and the protein was digested by trypsin. Tryptic peptides were enriched using anti-acetyllysine antibody agarose and the acetylated DRP1 peptides were analyzed using LC-MS/MS (Orbitrap Fusion MS; Thermo Fisher Scientific). For Western blotting experiments, mitochondrial and cytosolic fractions were separated from heart tissue samples and analyzed. It was found that lipid overload caused by a HFD in mice increased the levels of DRP1 predominantly in mitochondrial fractions compared to cytosolic fractions; however, the levels of fusion proteins mitofusin-1 (MFN1) and optic atrophy (OPA1; dynamin-like 120kDa protein) were unaltered. In the HFD group, DRP1 phosphorylation increased at serine 616 (S616) and decreased at serine 637 (S637) sites in whole heart samples as well as in mitochondrial fractions. Furthermore, DRP1 oligomerization increased in HFD hearts, mainly in mitochondrial fractions, and the GTPase-hydrolyzing activity of DRP1 was enhanced as well. The results from HFHC-fed rhesus monkeys echoed the data obtained from HFD-fed mice; DRP1 levels were up-regulated, as was phosphorylation at DRP1 S616, and phosphorylation was down-regulated at DRP1 S637. The DRP1 oligomerization and GTPase activity were increased in HFHC monkey hearts. Investigating the biochemical changes in HFD hearts revealed that the levels of total intracellular NAD^+^ (regulator of protein acetylation) were decreased as well as the levels of nicotinamide phosphoribosyltransferase, NAMPT, the enzyme related to NAD^+^ synthesis. Acetyl-CoA amounts in HFD hearts were enhanced. In addition, DRP1 acetylation was up-regulated in both HFD mice and HFHC monkey hearts in the mitochondrial fraction but not in the cytosolic fraction, suggesting that acetylation was associated with DRP1 translocation from the cytosol to the mitochondria. Thus, lipid overload promoted DRP1 acetylation. The following experiments in the culture of adult cardiomyocytes showed that under lipotoxicity caused by palmitate (0.3 mmol/L, 24 h), cytosolic NAD^+^ levels decreased and DRP1 alteration repeated the changes observed in HFD hearts. Palmitate caused mitochondrial fission, sensitivity to mitochondrial permeability transition pore (mPTP) opening and cardiomyocyte death. By supplementation of NAD^+^ into cardiomyocyte cultures (using NAD^+^ precursor nicotinamide ribose chloride or NAD^+^ precursor nicotinamide mononucleotide or overexpression of NAMPT), the increase in the DRP1 level was blocked, along with DRP1 S616 phosphorylation and DRP1 acetylation. MS-based proteomics identified DRP1 acetylation at lysine sites K75 and K642 in HFD hearts and no acetylated peptides in control CD mice hearts. Further observations of adult cardiomyocytes suggested that palmitate regulated DRP1 activity and stability by acetylation of DRP1 at the K642 site. Next, DRP1 activation was studied in terms of promoting lipid overload-induced cardiomyocyte death. Using co-immunoprecipitation, DRP1 interacted with VDAC1 (mitochondrial outer membrane protein and regulator of apoptosis), and this interaction was enhanced in HFD hearts. In summary, in HFD-fed mouse hearts, DRP1 levels were up-regulated and a HFD regulated DRP1 phosphorylation, mitochondrial translocation from cytosol, oligomerization and GTPase activity in mice hearts. Similarly, in HFHC diet-fed monkeys, lipid overload resulted in DRP1 activation and myocardial damage. It was suggested that NAD^+^-modulated acetylation of DRP1 regulated the DRP1 levels and activity. Furthermore, the K642 acetylation of DRP1 during lipid overload may be important for DRP1 activation, and lipid overload-induced cardiomyocyte death was mediated via DRP1 activity, DRP1 acetylation and VDAC1.

The review articles that have emerged over the past five years have reported the metabolic regulation of SIRT3, SIRT4 and SIRT5 in lysine acetylation, malonylation, succinylation and glutarylation [34,102]; the roles of PTMs in various diseases [103]; chemical and physiological features of mitochondrial acylation [33]; and molecular mechanisms of acetylation during regulation of fusion and fission in mitochondria [35].

### 4.2. Phosphorylation

Reversible phosphorylation is another PTM that has a crucial role in mitochondrial processes and functions. Recent progress in phosphoproteomic techniques has enabled many phosphorylated sites in the mitochondrial proteins to be identified. The constantly developing methods for mitochondrial isolation and fractionation, phosphopeptide/phosphoprotein enrichment and MS-based proteomics, with efficient and accurate analysis of accumulated data, have resulted in increased knowledge of the mitochondrial phosphoproteome. The studies confirmed that the impairment of mitochondrial phosphatases resulted in the dysfunction of the mitochondrial metabolism, implying the importance of phosphoproteome regulation for mitochondria homeostasis [29,104,105]. The extensive phosphorylation of IMM protein complexes and enzymes has been identified in functional mitochondria isolated from human muscle biopsies [104]. In [104], various techniques for phosphopeptide enrichment were implemented followed by LC-MS/MS identification of phosphorylated proteins. Thus, 77 mitochondrial proteins were identified with 155 phosphorylated sites containing 116, 23 and 16 phosphorylated serines, threonines and tyrosines, respectively. The mitochondrial phosphoproteins were associated, among others, with OXPHOS, the TCA cycle and lipid metabolism and they were the substrates for kinases A and C, casein kinase II and DNA-dependent kinase [104]. In 2009, the turnover of protein phosphorylation was examined in the mitochondrial matrix [106] by use of ^32^P protein labeling, and the experiments confirmed the existence of dynamic and extensive mitochondrial matrix phosphoproteomes in both the heart and liver.

The potential mechanism of microtubule-associated protein 4 (MAP4) phosphorylation in cardiomyocyte mitochondrial dysfunction has been reported using cardiac proteomics [12]. Based on previous results, MAP4 binding to microtubule is regulated via MAP4 phosphorylation [107], and the MAP4 phosphorylated sites S768 and S787 in humans (S737 and S760 in mice) controlled MAP4 detachment from microtubules [108]. After detachment, phosphorylated MAP4 is translocated to mitochondria during hypoxia in neonatal rat cardiomyocytes and it causes cardiomyocyte mitochondrial dysfunction, cardiac dysfunction and pathological remodeling [108]. In [12], differentially expressed proteins of aberrant MAP4 phosphorylation were examined in vivo by creating a mouse strain with hyperphosphorylated MAP4 at S737 and S760 sites (24-week-old; knock-in MAP4 KI) and comparing it to the WT control group. The proteins of heart tissue homogenates were trypsin-digested and tryptic peptides were labeled by iTRAQ reagents for comparative protein identification/quantification by a subsequent LC-MS/MS analysis (Easy nLC coupled to Q Exactive MS; Thermo Fisher Scientific, Waltham, MA, USA). The MASCOT search engine v. 2.5 in the Proteome Discover 2.1 platform (Thermo Electron, Waltham, MA, USA) was used against UniProt mouse database (*p* ≤ 0.05 was statistically significant, FC ≤ 0.833 or FC ≥ 1.2). Out of 3812 proteins identified, 72 proteins were differentially expressed with 12 proteins up-regulated and 60 proteins down-regulated in the MAP4 KI group compared to the WT group. Using a GO database analysis of differentially expressed proteins, the most enriched proteins were related to GTP binding, guanyl ribonucleotide binding and guanyl nucleotide binding. The pathway analysis of differentially expressed proteins identified several cardiomyopathy-related pathways with hypertrophic cardiomyopathy as one of the most enriched pathways, implying the correlation of MAP4 phosphorylation with this disease. The MS data were confirmed by Western blotting for the proteins that were involved in regulation of mitochondrial function, i.e., mitochondrial ubiquitin ligase activator of NFKB (MUL1), growth arrest and DNA damage-inducible gamma interaction protein 1 (GADD45GIP1) and NADH-ubiquinone oxidoreductase 75 kDa subunit (NDUFS1). GADD45GIP1 levels were higher, whilst MUL1 and NDUFS1 levels were significantly lower in the MAP4 KI group compared to the WT control. In summary, this work identified proteins that were differentially expressed in heart tissue as a result of MAP4 phosphorylation, and it contributed to the knowledge on molecular mechanisms of MAP4 phosphorylation-induced mitochondrial dysfunction [12].

A comprehensive dataset of dynamic changes in the proteome and phosphoproteome has been compiled in mouse hearts during postnatal development using spike-in SILAC [109] quantification and LC-MS/MS [110]. In [110], neonatal mice hearts were extracted at 2 (P2), 10 (P10) and 20 (P20) days after birth as well as age-matched hearts of mice labeled with a ^13^C_6_ lysine (heavy; Lys6)-containing diet which were used as spike-in heavy standards for quantification of non-labeled experiments. The samples of heart tissues were subjected to 1-DE, and each lane was cut into six to twelve pieces. Equal proportions of Lys6-labelled and non-labelled protein bands were combined, in-gel digested using Lys C and the resulting peptides were analyzed by LC-MS/MS. A total of 24,366 phosphorylated sites were mapped to 4953 identified proteins; the majority of them were single phosphorylated peptides. A total of 84.3% of phosphorylated peptides were serine phosphorylated, 15.3% were threonine phosphorylated and 0.4% were tyrosine phosphorylated. The significant fold changes (FC > 1.5; FDR < 0.05) in the expression of phosphorylated sites measured at time points P2 and P20 (both related to time point P10) showed 89 and 573 phosphopeptides differently regulated, respectively. The proteins that were annotated as mitochondrial included 495 phosphorylated sites. For example, subunits of the OXPHOS system exhibited an alteration in the expression of phosphorylated sites during development, and increased phosphorylation was identified for MIC60 (MICOS component subunit) at S113, S128 and S446 sites during development from P10. Furthermore, association of protein PGC-1- and ERR-induced regulators in muscle 1 (PERM1) to the outer mitochondrial membrane was detected as well as its upregulation during heart development from P2 to P20. PERM1 was highly phosphorylated with 34 phosphorylated sites identified. It was confirmed that PERM1 phosphorylation at S347, S358, S369 and S380 was mediated by casein kinase 2 (CK2), and phosphorylation regulated the stability of PERM1. Furthermore, to calculate the rate of Lys6 incorporation into newly synthesized proteins, mice fed with a Lys6-containing diet for 1–4 weeks were examined by LC-MS/MS and the ratios between non-labeled and Lys6-labelled peptides in heart tissues were calculated. PERM 1 showed a faster Lys-6 incorporation (6.4 days) compared to other mitochondrial proteins such as VDAC2, cytochrome c oxidase subunit 5B (COX5B) and heat shock protein 60 (HSP60) (average of 13 days). Furthermore, the experiments on PERM1-deleted mitochondria showed that mitochondrial proteins that mediated transport of amino acids and carnitine were down-regulated and that the levels of lipids, amino acids and acylcarnitines were changed. Thus, the authors concluded that mitochondrial PERM1 was crucial for lipid and amino acid homeostasis and contributed to the regulation of energy metabolism in mitochondria.

**Figure 1:**
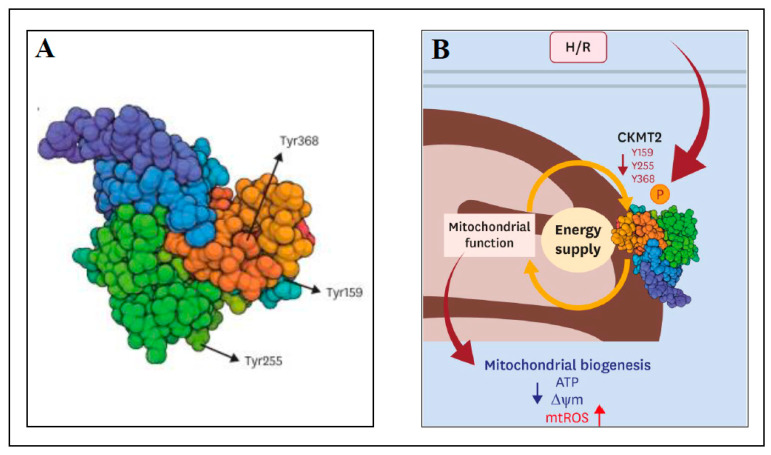
Cardioprotection against H/R injury by tyrosine phosphorylation of human CKMT2. (**A**) The structure of human CKMT2 with three phosphorylated sites of tyrosine (Y159, Y255 and Y368); (**B**) A scheme indicating the tyrosine residues affected during H/R, which led to modulation of CKMT2 activity and mitochondrial function. H/R—hypoxia/reoxygenation, ATP—adenosine triphosphate, ∆ψm—mitochondrial membrane potential, mtROS—mitochondrial reactive oxygen species. More details can be found in the text. This figure was adapted from ref. [111].

The aim of the next study was to identify and characterize novel phosphorylated proteins in mitochondria that could be efficient in the prevention of I/R injury [111]. Sprague Dawley rats were used as the models of normal perfusion (control; 20 min), ischemia only (20 min), I/R (20 min ischemia and 20 min reperfusion) and ischemic preconditioning (IPC; 20 min IPC and 20 min perfusion) using Langendorff apparatus. Usually, IPC includes brief periods of ischemia (hypoxia) and reperfusion (reoxygenation) prior the ischemic insult. In [111], the phosphoproteins in rat hearts were analyzed using 2-DE and MALDI-TOF MS. A comparison of perfused rat hearts to IPC hearts found 54 proteins exclusively detected in IPC heart samples with 515 phosphorylated sites. Mitochondrial creatine kinase S-type (CKMT2) was among the proteins that were phosphorylated during IPC with the tyrosine Y368 site phosphorylated in IPC and perfusion samples, and dephosphorylated in ischemia only and I/R samples. To test the previous hypothesis that CKMT2 overexpression can result in recovery of cardiac pathologies [112], the following experiments were performed with human cardiac ventricular cell line AC16 transfected by MTS-GFP or CKMT2-GFP and subjected to normoxia (normal level of oxygen) or 18 h hypoxia/2 h reoxygenation (H/R) [111]. In H/R conditions, CKMT2 overexpression resulted in increased cell viability compared to control MTS-GFP, preservation of mitochondrial membrane potential, and a reduction in ROS production (independently of ATP production), implying cardioprotection against H/R. Mutagenesis of the phosphorylated sites of CKMT2 (Y159A, Y255A and Y368A; substitution of tyrosine for alanine) decreased cell viability and enhanced ROS generation under H/R, thus abolishing the cardioprotective mechanism. The mutation of phosphorylated sites of CKMT2 in transfected AC16 cells under normoxia decreased the expression of peroxisome proliferator-activated receptor γ coactivator-1α (PGC-1α; regulator of OXPHOS and FAO systems) by about 20–50% and decreased its expression even more (70%) after H/R. Similar decreases were observed for transcription factor ERRα (estrogen-related receptor alpha) and PHB1 (prohibitin 1; regulator of mitochondrial stabilization). In summary, mutations in phosphorylated sites of CKMT2 caused decreases in CKMT2 activity and mitochondrial dysfunction under H/R and thus, the cardioprotective function of CKMT2 was compromised. The data pointed to a novel regulatory role of CKMT2 in cardioprotection after I/R and H/R injury. Figure 1 shows the structure of human CKMT2 with novel phosphorylated tyrosine sites Y159, Y255 and Y368 (Figure 1A) and the scheme of cardioprotection against H/R injury by phosphorylation of CKMT2 (Figure 1B).

The phosphorylated mitochondrial proteins involved in the nitric oxide (NO)/protein kinase G (PKG)/mPTP pathway were investigated in a rat cardiac ischemia model [46]. Previously, a NO-triggered cardioprotective effect has been reported during ischemia injury; however, the precise molecular mechanism was unknown. In addition, the pretreatment of hearts with NOC-18 (donor of NO) is known to reduce the effect of ischemia and I/R injury in mitochondria [113], and this prevention is regulated by PKG and protein kinase C (PKC) that are activated by NO and act against mPTP opening [113]. In [46], the hearts isolated from Wistar rats were subjected to ischemia or perfused by NOC-18 (1 µM, 4 min) before stop-flow global ischemia (30 min), and the mitochondria were isolated from homogenates by differential centrifugation. The mitochondria were lysed and the proteins were subjected to 2-DE with Pro-Q diamond gel staining (for phosphoproteins) and SYPRO Ruby staining (for total proteins). The protein spots from the gels were excised and in-gel trypsin-digested prior to identification by LC-MS/MS (nanoAcquity UPLC system coupled to HDMS Synapt G2 MS (Waters Corp., Wilmslow, UK) via a nano-ESI7 tip emitter (New Objective, Littleton, MA, USA)). For phosphoprotein enrichment experiments, the mitochondria were lysed and the phosphoproteins were enriched using Pro-Q Diamond phosphoprotein-binding beads, digested by trypsin and analyzed by LC-MS/MS. The NOC-18-pretreated ischemic hearts and ischemic hearts yielded a total of eighty protein spots, with six protein spots showing an FC of > 1.5 between these two conditions. MS identified ATP synthase subunit alpha (ATP5A1) with increased expression in NOC-18-pretreated ischemic hearts. The subsequent global proteomic analyses, which included phosphoprotein enrichment, exhibited an alteration in phosphorylated proteins of NOC-18-pretreated ischemic hearts compared to ischemia group, i.e., nine proteins were associated with the mitochondrial membrane and an additional ten mitochondrial matrix proteins were significantly up-regulated, whilst one protein was significantly down-regulated. Among them were the up-regulated ATP5A1 and adenine nucleotide (ADP/ATP) translocase 1 (SLC25A4), which are known to be the components of mPTP. Furthermore, the experiments were performed with normal mitochondria isolated from non-ischemic rat hearts and treated directly by PKG for 15 min in order to evaluate the similarities in mitochondrial phosphoproteome with the group treated by NOC-18. The phosphoproteins from PKG-treated mitochondria and separated on 2-DE gels showed no alteration compared to phosphoproteins from the control without PKG treatment. However, the alteration was detected in whole mitochondrial proteome treated by PKG compared to the control without PKG treatment, i.e., 2-oxoglutarate dehydrogenase complex (DLST), malate dehydrogenase (MDH) and the spot consisting of IDH2 and cytochrome b-c1 complex subunit 2 (UQCRC2) were down-regulated, whilst the gel spot containing VDAC1, VDAC3, 2,4-dienoyl-CoA reductase (DECR1) and D-beta-hydroxybutyrate dehydrogenase 1 (BDH1) was up-regulated compared to the control. The protein phosphorylation pattern detected in the hearts pretreated with NOC-18 did not replicate the pattern detected in isolated PKG-treated normal mitochondria. Thus, mitochondrial proteins related to OXPHOS and FAO systems might be regulated via other NO-mediated pathways as some beneficial activity of NO has been reported to be independent of PKG [113]. It was concluded that the treatment of rat hearts with NOC-18 prior to ischemia caused an alteration in mitochondrial phosphoproteome, and several differentially phosphorylated proteins were considered to mediate mPTP opening and the redox state.

In addition, methods have been published that were applicable for large-scale analysis of mitochondrial phosphoproteome in various cells and tissues [114]. They include the protocols for isolation of mitochondria and its purity screening, gel-based analysis (2-DE with phosphoprotein staining followed by MS identification) and gel-free analysis (in solution protein digestion followed by identification of phosphorylated residues by MS). Other articles have been published that have reviewed the PTMs of mitochondrial outer membrane proteins, such as phosphorylation, acetylation, nitration and carbonylation [31,115]; mitochondrial phosphoproteomics in mammalian tissues with an impact on large-scale studies of mitochondria from human skeletal muscle [28]; and protein phosphorylation within mammalian mitochondria including the known functions of mitochondrial-resident phosphatases [29]. Recently, an article has reviewed the PTMs of mitochondrial proteins that were the components of mPTP [27], such as cyclophilin D (CYPD), VDAC, the FoF1 ATP synthase complex (FoF1 ATPase), adenosine nucleotide translocase (ANT), CK, phosphate carrier (PIC), hexokinase (HK) and the 18 kDa translocator protein (TSPO), and the relation of these PTMs (phosphorylation, acetylation, oxidation and oxidative PTMs (S-nitrosylation, S-glutathionylation, nitration, deamination and ubiquitylation)) to cardiac diseases.

Table 2 includes the studies of mitochondrial protein PTMs (acylation and phosphorylation) in various cardiovascular diseases and in other heart conditions that were discussed in detail in this section.

## 5. Mitochondrial Dysfunction and Its Implication for Cardiovascular Diseases

The selected proteomic studies published in the last five years are overviewed with a focus on two heart diseases, myocardial I/R injury and cardiomyopathy. Papers that include proteomics of isolated mitochondria as well as proteomics of whole cell lysates with mitochondrial protein identification are discussed. Although one study targeted PTM (tyrosine nitration) changes during I/R, it was included in this section.

### 5.1. Ischemia-Reperfusion Injury

An acute myocardial infarction (AMI), commonly termed as a heart attack, is an ischemic event that creates a high danger of developing HF. In an AMI, blood flow to the heart is suddenly restricted or blocked and it prevents the heart from receiving enough oxygen. This causes a necrosis of the tissue within at-risk myocardium and the final loss of functional tissue (infarct size) delineates the future clinical outcomes. The early blood restoration after the ischemic period, i.e., reperfusion, can prevent the increase in the infarct size of the ischemic tissue; however, it can have further negative consequences such as arrhythmias, dysfunction of cardiac contractility or even cell death [21,116]. During I/R, mitochondria function and integrity are compromised, with increased ROS/RNS generation and calcium overload [117], decreased ATP production, damage to the ETC and transport of pro-apoptotic factors from the mitochondria into the cytosol (e.g., apoptosis inducing factor (AIF) and cytochrome c [118,119]). Timely removal of damaged mitochondria via mitophagy is critical [39]. However, the mechanisms triggered during ischemia often prevent defected mitochondria to be released from myocytes and thus a proper cellular function is jeopardized [120,121]. It has been shown that conditioning of the heart can reduce myocardial injury either by pharmaceuticals or mechanically, i.e., by brief periods of ischemia (hypoxia) and reperfusion (reoxygenation), either prior to lethal myocardial ischemia (ischemic preconditioning; IPC) or after myocardial ischemia (post-conditioning) during the first minutes of reperfusion [21,22,122,123,124].

Recently, a proteomic approach has been applied to evaluate mitochondrial and cardiomyocyte injury as a result of ETC damage during cardiac I/R (mouse C57BL/6; 25 min global ischemia and 30 min reperfusion) [120]. Prior to ischemia, amobarbital was used as a reversible blocker of ETC at complex I (2 mM; 1 min), and mitochondria from untreated mouse hearts and mouse hearts treated by amobarbital were isolated after reperfusion. The mitochondrial proteins were digested and the resulting tryptic peptides were analyzed and quantified by an LC-MS/MS system (Shimadzu LC-20AD-HPLC (Tokyo, Japan) and QTrap5500-ESI MS (AB Sciex, Toronto, ON, Canada)) using iTRAQ labeling. Without treatment, the levels of multiple enzymes involved in the FAO pathway, the TCA cycle and the ETC decreased. On the contrary, a reversible blockade of ETC by amobarbital treatment prevented these decreases, reduced the mitochondrial damage and resulted in decreased cardiac injury and protected respiration. In summary, in addition to ETC damage, multiple pathways of the intermediary metabolism were affected after I/R, and these changes could be protected by modulation of ETC function [120].

Calpains (CPN1 and CPN2) are the proteins of the calcium-dependent, non-lysosomal cysteine proteases family and their activities are dependent on the intracellular calcium concentration. They have been involved in mitochondrial injury and implicated in the development of, e.g., myocardial infarction (MI), after I/R. Historically, they are known to be cytosolic proteins. However, both mitochondrial calpains (mCPN1 and mCPN2) have been found in various locations in cardiac mitochondria compartments dependent on their functions in various pathological conditions [125,126,127]. The activation of mCPN1 and mCPN2 by calcium overload during I/R resulted in the cleavage of cytosolic and mitochondrial proteins and contributed to mitochondrial damage. For example, mCPN1 mediated AIF cleavage and the release of AIF from mitochondrial IMS (mice hearts) causing caspase-independent apoptosis [118]. mCPN2 has been shown to induce the inactivation of complex I of the ETC via truncation of the ND6 subunit, and mCPN2 contributed to mPTP opening either after subjecting isolated mitochondria to 500 µM of Ca^2+^ or during I/R in rat hearts [128]. Recent work has used a proteomic approach to determine the protein targets of mitochondrial CPN1 and CPN2 during I/R [119]. Both CPN1 and CPN2 contain calpain small subunit 1 (CPNS1), which is responsible for CPN1 and CPN2 activities as confirmed by genetic deletion of CPNS1 [129]. In [119], isolated hearts from WT and cardiomyocyte-specific CPNS1-deleted mice were subjected to 25 min ischemia followed by 30 min reperfusion. Compared to the WT, the CPNS1-deleted mice showed decreased cytosolic and mCPN1 activation, decreased cardiac injury (decreased infarct size), improved mitochondrial function (decreased inclination to mPTP opening and improved oxidative phosphorylation), decreased production of ROS (H_2_O_2_) and lower amounts of cytochrome c and AIF were released outside of mitochondria. In addition, quantitative proteomics of isolated mitochondria identified up-regulation of proteins α-crystallin B chain, as well as paraplegin and sarcalumenin (the proteins regulated calcium homeostasis in mitochondria) and the proteins related to complex III in CPNS1-deleted mice. The proteomic analysis included separation of proteins by SDS-PAGE, in-gel digestion of the bands and protein identification using a UltiMate 3000 UHPLC system (Thermo Fisher Scientific, Dreieich, Germany) interfaced with an Orbitrap Fusion Lumos Tribrid MS (Thermo Fisher Scientific, Dreieich, Germany) performed in DDA acquisition mode. The data were analyzed by Thermo Scientific Proteome Discoverer (PD) software V2.3 against the UniProt mouse protein database (16,996 entries) [119].

Although nitrotyrosine formation in reversible postischemic contractile heart dysfunction (known as myocardial stunning) has been evaluated previously using immunohistochemistry and Western blotting [130], a recent study has applied comparative proteomics to investigate the molecular mechanism of myocardial stunning and tyrosine nitration (3-nitrotyrosines) of mitochondrial proteins of stunning hearts [131]. Rat hearts were subjected to 20 min no-flow ischemia or 20 min global ischemia with a subsequent 30 min reperfusion. Control hearts were perfused for 50 min without ischemia. The samples of isolated mitochondria from male Wistar rat heart homogenates were subjected to 2-DE followed by Western blotting with anti-3-nitrotyrosine monoclonal antibody. Comparing the results of the membranes and gels, the gel spots of interest were analyzed using MALDI-TOF/TOF MS (Ultraflex III; Bruker Daltonics, Germany) after their excision and protein in-gel digestion. Eight mitochondrial proteins were detected as significantly tyrosine nitrated after I/R, i.e., aconitate hydratase (ACO2), dihydrolipoyl dehydrogenase (DLD), electron transfer flavoprotein-ubiquinone oxidoreductase (ETFDH), isovaleryl-CoA dehydrogenase (IVD), ATP synthase subunit β (ATP5B), β-enolase (ENO3), long-chain specific acyl-CoA dehydrogenase (ACADL) and CKMT2. These results were in accordance with immunofluorescence analyses in which protein tyrosine nitration was not significantly increased in ischemic myocardium; however, a significant increase in both protein tyrosine nitration and the number of 3-nitrotyrosine positively stained cardiomyocytes was detected in reperfused hearts. Compared to control hearts, protein tyrosine nitration was higher in hearts after I/R, although the relative abundances of nitrated proteins were lower, implying extensive mitochondrial protein nitration after reperfusion. The authors deduced that enhanced tyrosine nitration of mitochondrial proteins in stunned myocardium could result in premature proteolytic degradation and thus in reduced amounts of mitochondrial enzymes. Although only a few proteomic studies have been published to date dealing with protein nitration after myocardial I/R, 10 mitochondrial proteins have been found as nitrated after 60 min ischemia and 60 min reperfusion [132] and 13 mitochondrial proteins have been identified with enhanced nitration in a guinea pig heart model after 30 min ischemia followed by 10, 30 and 60 min of reperfusion [133]. The list of proteins identified in [132] differed from the proteins detected in [133]. Only the proteins DLD and ATP5B were common to all three studies [131,132,133].

Sestrin2 is an essential protein that regulates the cell response to various stresses [134] and it is down-regulated during aging of the heart, which reduces the heart’s resistance to damage induced by I/R [135]. Targeted proteomics has been used to analyze the effect of sestrin2 in three groups of C57BL/6J mice: those subjected to I/R at the ages of 3–6 months and 24–26 months and sestrin2-knockout (KO) mice at the of age 3–6 months. Extracted proteins from left heart ventricle tissue were immunoprecipitated with sestrin2 antibody, digested and then analyzed by LC-MS/MS (Thermo Easy nLC 1200 coupled to Thermo Q-Exactive-HF MS), with three biological replicates for each group. To better characterize the changes in the proteins that were identified as a response to I/R, the proteins were subjected to IPA (Qiagen; Germany), and the pathways that involved sestrin2, associated proteins and upstream transcriptional regulators were analyzed. It was found that mice aged 24–26 months and sestrin2-KO hearts exhibited damage to mitochondria complex I and complex II activities as well as impaired respiration rates compared to young hearts (3–6 months). Furthermore, sestrin2 translocation into mitochondria was detected, as well as its interaction with OXPHOS components, in response to I/R stress. In addition, the data revealed that a sestrin2 deficiency contributed to diastolic dysfunction and that sestrin2 has a key role in maintaining mitochondrial functional integrity via modulation of mitochondrial biosynthesis and assembling of OXPHOS complexes. Moreover, sestrin2 was associated with FAO components during I/R and it has an influence on isocitrate dehydrogenase and pyruvate dehydrogenase activities. The authors summarized that the decrease in sestrin2 in aged hearts led to the decrease in mitochondrial functional integrity after I/R. Sestrin2 acted as a scaffold protein that contributed to mitochondrial integrity and it was a crucial protein for protection of the heart against I/R via substrate metabolism regulation [135].

Comparative proteomics has been used to identify differentially expressed proteins in isolated rat hearts subjected to normal, I/R and diazoxide post-conditioning (D-post) [22]. The I/R and D-post hearts were subjected to 40 min ischemia followed by reperfusion for 60 min in the I/R group, and by reperfusion with diazoxide (50 µM) for 2 min followed by reperfusion for 58 min in D-post group. Diazoxide is one of a number of drugs (mitochondrial ATP-sensitive potassium channel openers) that have myocardium protective functions [136]. Previously, it has been shown that D-post protected myocardia against I/R injury [137] via opening of the ATP-dependent potassium channel located in the IMM [138]. In [22], 2-DE followed by MALDI-TOF/TOF MS (Ultraflex III, Bruker Corp.) was applied to identify differences in the expression profiles of the mitochondrial proteins after mitochondria extraction from ventricular tissues by differential centrifugation with a density gradient. Four proteins were up-regulated in D-post samples compared to I/R samples, NADH dehydrogenase (ubiquinone) flavoprotein 1 (NDUFV1), NDUFS1, ATP synthase alpha subunit and 2-oxoglutarate dehydrogenase (OGDH). These results were validated for NDUFV1, NDUFS1 and OGDH proteins by Western blotting. In conclusion, these particular mitochondrial proteins may be the part of the protective mechanism in D-post condition and the therapeutic targets in cardiac I/R injury [22].

The molecular mechanism of oxidative injury to mitochondria complex III after cardiac ischemia has been examined recently [139], providing a better understanding of how the impairment of complex III contributed to mitochondrial dysfunction of post-ischemic hearts. Complex III (cytochrome c oxidoreductase) in mitochondrial ETC is the catalyst of ubiquinol (QH_2_) oxidation via the reduction of ferricytochrome c in the Q-cycle. In this cycle, complex III is involved in the pathway of electron transfer from QH_2_ to the Rieske iron-sulfur cluster (2Fe-2S) and to heme c_1_, and this is controlled by the groups of heme b_L_ (low potential) and b_H_ (high potential) and by two semiquinones. Complex III’s catalytic mechanism contributes to ATP synthesis and generation of endogenous superoxide anion radicals (^.^O_2_^−^). In [139], a rat heart model of acute myocardial infarction was utilized by placing a suture on the left anterior descending coronary artery (30 min ischemia) followed by 24 h reperfusion. The mitochondrial proteins in native PAGE gel bands from at-risk myocardial tissues of sham, ischemia and I/R were enzyme digested (trypsin/LysC or chymotrypsin) and the samples with tryptic or chymotryptic peptides were subjected to nLC coupled with Orbitrap Fusion MS/MS (Thermo Scientific). For protein quantification, label-free quantitation was applied using MaxQuant software. An MS analysis identified (i) cleavage of the covalent thioether linkage between cysteine residue C_122_ of cytochrome c_1_ and C_125_ of heme c_1_ in complex III of I/R samples; this was not observed in sham or ischemic heart samples; (ii) oxidative modification of complex III by cysteine sulfonation/sulfination (SH group conversion to the SO_3_H/SO_2_H group; mass shift of 48 Da/32Da) after I/R in contrast to sham and ischemia samples; specifically, a significant increase in cysteine sulfonation and/or sulfination of the metal binding sites of the Rieske iron-sulfur cluster (cysteine residues C_217_ and C_236_), cytochrome c_1_ (C_140_ and C_220_) and other subunits of complex III (hinge protein (C_65_), core 1 (C_69_, C_268_, C_380_, C_410_, C_445_ and C_453_) and core 2 (C_191_)), and (iii) specific sulfonation of C_236_ caused damage to the metal binding of the Rieske iron-sulfur cluster by blocking oxidation of ubiquinol and thus electron transfer from ubiquinol to heme c_1_ through the (2Fe-2S) cluster. Since the C_236_ site of the Rieske iron-sulfur cluster is located inside of the cluster and it is not accessible to oxidation (as confirmed experimentally using cumene hydroperoxide), it was implied that C_236_ is oxidized by ROS that are generated locally under stress created by I/R. The sulfonated cysteine sites of cytochrome c_1_ and the hinge protein are confined in the IMS of mitochondria, and core 1 and core 2 sulfonated cysteine sites are positioned in the mitochondrial matrix compartments. This fact further supported the idea that complex III residing in the IMM released ROS to both sides of the IMM during reperfusion [139]. In summary, I/R-induced stress resulted in both heme c_1_ destruction by overproduction of ROS in the post-ischemia and in the enhanced oxidative sulfonation of the Rieske iron-sulfur cluster of complex III. These events contributed to increased formation of O_2_^−^, which worsened I/R injury.

**Figure 2:**
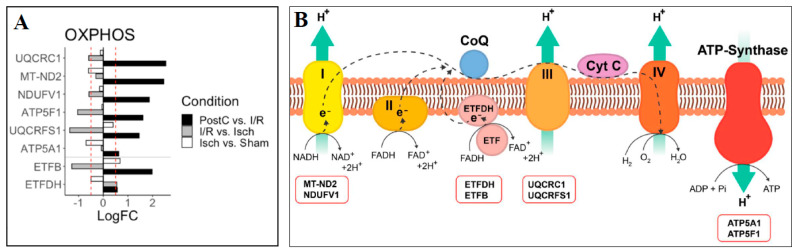
Oxidative phosphorylation (OXPHOS)related differentially regulated proteins. (**A**) The changes in expression of proteins (Log FC) during ischemia (Isch), ischemiarevascularization (I/R) and ischemic post-conditioning (PostC); red dashed lines indicate absolute Log FC > 0.5; (**B**) A scheme showing ETC complexes of OXPHOS and FoF1 ATP synthase complexes (ATP-Synthase) with identified differentially regulated proteins (red boxes). UQCRC1—cytochrome b-c1 complex subunit 1, MTND2—NADH-ubiquinone oxidoreductase chain 2, NDUFV1—NADH dehydrogenase [ubiquinone] flavoprotein 1, ATP5F1−ATP synthase beta subunit, UQCRFS1—cytochrome b-c1 complex subunit Rieske, ATP5A1—ATP synthase subunit alpha, ETFB−electron transfer flavoprotein subunit beta and ETFDH—electron transfer flavoprotein-ubiquinone oxidoreductase. More details can be found in the text. This figure was adapted from ref. [21].

Recent work has examined the mitochondrial proteome in a pre-clinical model of ischemia (90 min of left anterior descending (LAD) coronary artery balloon occlusion), ischemia-revascularization (90 min LAD occlusion followed by 2.5 h of revascularization (balloon disinflation; restoration of perfusion to myocardium after ischemia [140])) and ischemic post-conditioning (90 min LAD occlusion with subsequent post-conditioning and 2.5 h of revascularization) in a swine model of AMI [21]. The post-conditioning included six cycles of 20 s of revascularization and 20 s of ischemia on the onset of 2.5 h revascularization. Protein extracts were prepared from the heart tissues of the inner border zone of the area at risk by homogenization in urea/thiourea buffer. The proteins from the extracts were subjected to 2-DE, the gels were fluorescent labeled (Flamingo Fluorescent gel stain) and the spot variations among the samples of sham, ischemia, revascularization and post-conditioning were determined. Proteins from the spots of interest were identified by MALDI-TOF/TOF MS (Bruker Daltonics, Billerica, MA, USA). Differentially expressed proteins (logFC > 0.5) were further examined using a protein–protein interaction (PPI) network analysis and Gene Ontology for determination of biological processes, functions and biological pathways. At least 26 mitochondrial proteins were identified as differentially expressed across all conditions with eight proteins involved in the TCA cycle, six proteins belonging to ETC complexes I and III and the FoF1 ATP synthase complex and seven proteins with non-metabolic functions. During ischemia, the TCA cycle enzymes DLD and succinyl-CoA ligase (SUCLA2) were down-regulated with no recovery during revascularization. Moreover, after revascularization, other proteins of the TCA cycle were down-regulated, e.g., isocitrate dehydrogenase subunit alpha (IDH3A) and IDH2. Only OGDH was up-regulated after revascularization. After post-conditioning, IDH3A was recovered and the enzymes involved in the TCA cycle progression were highly up-regulated, implying that TCA cycle progression is promoted by a post-conditioning event. Furthermore, up-regulation was observed for NADH-ubiquinone oxidoreductase chain 2 (MTND2) and NDUFV1 of complex I of ETC and for subunit UQCRC1 of complex III of ETC during post-conditioning compared to the sham group. Up-regulation of mitochondrial inner membrane protein OXA1L (the protein that mediates insertion and proper assembling of integral proteins at IMM) was also up-regulated. Subunit UQCRFS1 of complex III of ETC and the subunits ATP5A1 and ATP5F1 of the FoF1 ATP synthase complex remained unchanged during post-conditioning compared to sham, and down-regulated compared to revascularization. These facts confirmed the crucial roles of ETC complexes in cardioprotection during post-conditioning. ETFDH and the electron transfer flavoprotein subunit beta (ETFB), which create a link between the oxidative phosphorylation (ETC complexes) and FAO, were up-regulated during post-conditioning; thus, a more significant role of FAO in metabolism can be suggested after ischemia, and cardiac metabolism is implied as an important factor in heart protection. The non-metabolic proteins previously related to cardioprotection, DJ-1 (PARK7) and VDAC2, were up-regulated during post-conditioning compared to sham, along with mitochondrial stress-70 protein (HSPA9; mortalin), which is essential for DJ-1 mitochondrial importation [21]. This confirmed a protective response mediated by VDAC2 and DJ-1. A PPI network analysis showed that transcription factors CTCF, NR3C1 and NRF1 associated with the proteins identified in the post-conditioning group could be future targets for heart protection [21]. Figure 2 shows OXPHOS-related differentially regulated proteins during ischemia (Isch), ischemia-revascularization (I/R) and ischemic post-conditioning (PostC) (Figure 2A) and the scheme of ETC complexes of OXPHOS and FoF1 ATP synthase complex, with differentially regulated proteins identified in red boxes (Figure 2B).

### 5.2. Cardiomyopathy

Cardiomyopathy is a heart disorder in which the heart’s ability to pump blood to the rest of the body becomes restricted, which can lead to arrhythmias and eventually to HF. It affects the heart muscle, which can become stiff (restrictive cardiomyopathy), enlarged (dilated cardiomyopathy) and thickened (hypertrophic cardiomyopathy) and it can cause scar tissue.

**Figure 3:**
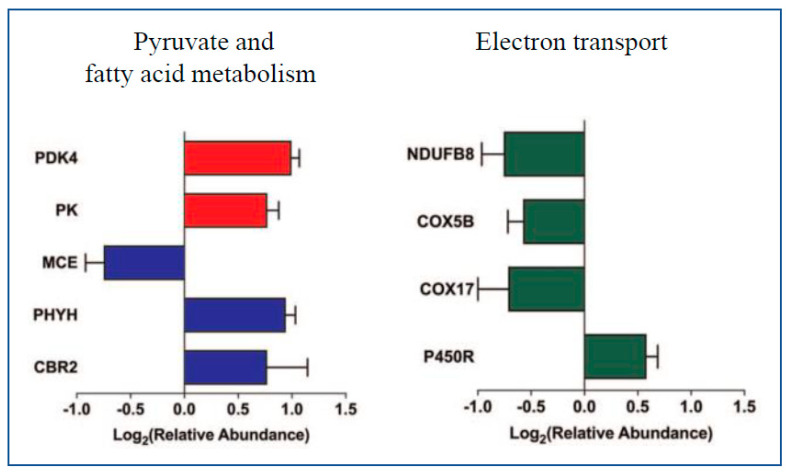
The mitochondrial proteins of isolated cardiac mitochondria from septic (CLP) mice involved in pyruvate metabolism (red), fatty acid metabolism (blue) and electron transfer (green) with altered expression. CLP—cecal ligation and puncture; Log2—logarithm of expression ratios between CLP and sham mice for the proteins with *p*-values < 0.05; PDK4—pyruvate dehydrogenase kinase 4, PK—pyruvate kinase, MCE—methylmalonyl-CoA epimerase, PHYH—phytanoyl-CoA dioxygenase, CBR2—carbonyl reductase (NADPH) 2, NDUFB8—NADH dehydrogenase (ubiquinone) 1 beta subcomplex subunit 8, COX5B—cytochrome c oxidase 5B, COX17—cytochrome c oxidase copper chaperone and P450R—NADPH-cytochrome P450 reductase. This figure was adapted from ref. [141].

The study of mitochondrial dysfunction and the profile of the mitochondrial proteome in polymicrobial sepsis-induced cardiomyopathy has been examined recently [141]. Septic cardiomyopathy can be described as reduced contractility of the left ventricle associated with left ventricle dilatation with/without right ventricular dysfunction [142]. In [141], proteins of mitochondria isolated from mice hearts of the sham group and of a model of a sepsis surgery group (cecal ligation and puncture; CLP) were compared. The proteins were processed by trypsin digestion and separated using nano-ACQUITY UPLC (Waters Corporation) coupled to an Orbitrap Fusion Lumos Tribrid MS (Thermo Scientific). Protein label-free quantification was performed using the accurate mass and retention time cluster quantification algorithm (Minora; Thermo Scientific). Out of 665 proteins identified as mitochondrial and/or metabolism related, 35 proteins were differentially expressed between the sham and the CLP group. One of the mechanisms considered during sepsis is dysfunction in mitochondrial bioenergetics [143]. Indeed, this corresponded to the changes observed in the mitochondrial proteome, as several of the 35 detected proteins were involved in pyruvate and fatty acid metabolism, the ETC system, lactate production and the integrity of mitochondrial membrane. Related to pyruvate metabolism and fatty acid metabolism, pyruvate dehydrogenase kinase 4 (PDK4), pyruvate kinase (PK), phytanoyl-CoA dioxygenase (PHYH) and carbonyl reductase (NADPH) 2 (CBR2) were up-regulated, whilst methylmalonyl-CoA epimerase (MCE) was down-regulated in the CLP group compared to sham. Associated with the ETC system, NADH dehydrogenase (ubiquinone) 1 beta subcomplex subunit 8 (NDUFB8) of complex I, cytochrome c oxidase 5B (COX5B) of complex IV and cytochrome c oxidase copper chaperone (COX17), which is essential for complex IV, were down-regulated in the CLP group. The enzyme NADPH-cytochrome P450 reductase (P450R) was up-regulated in CLP mice compared to sham. In addition, real-time qRT-PCR (quantitative reverse transcription PCR) validated the MS results for PDK4 expression in CLP isolated mitochondria as well as CLP heart tissue homogenate (6.8-fold increase compared to sham). Mitochondrial kinase PDK4′s function is to phosphorylate pyruvate dehydrogenase (PDH) and inhibit the PDH enzyme function [144]. Western blotting of the whole heart tissue showed a significant increase in PDH phosphorylation at S_293_ in CLP hearts. Moreover, a considerable decrease in PDH activity (PDH activity kit; Abcam) was observed in CLP hearts compared to sham, and the mitochondrial oxygen consumption rate (OCR; XF24 plate of Seahorse assay) in CLP hearts was decreased as well. Thus, cardiac dysfunction induced by sepsis was connected to inhibition of mitochondrial PDH and to compromised pyruvate-fueled oxidative respiration in the mice myocardium. In summary, this work on the sepsis myocardium identified molecular remodeling events that showed the functional relation between PDH inactivation and pyruvate/malate-based OCR without detection of any dysfunction of ETC complexes I, II and IV [141]. Figure 3 illustrates the mitochondrial proteins of isolated cardiac mitochondria from CLP (cecal ligation and puncture)-induced septic mice that were involved in pyruvate metabolism (red), fatty acid metabolism (blue) and electron transfer (green) with altered expression.

**Figure 4:**
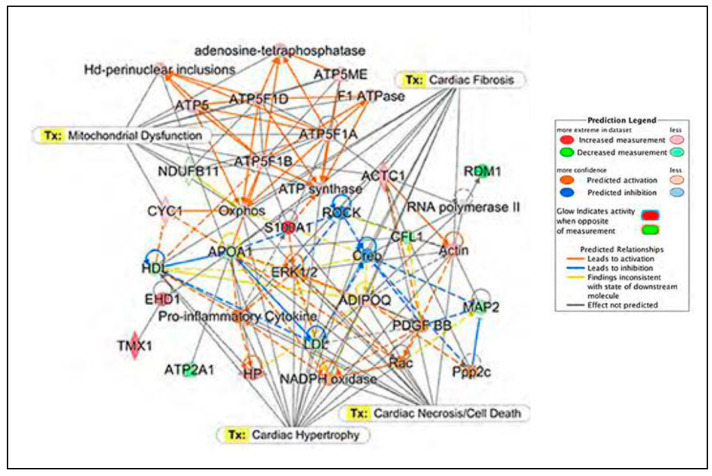
IPA analysis of differentially expressed proteins in diabetic mice with predicted biological processes. Differentially expressed proteins in diabetic mice (compared to WT) were engaged in biological processes such as mitochondrial dysfunction, cardiac fibrosis, cardiac hypertrophy and cardiac necrosis/cell death. The color nodes in the network of graphical relations between proteins imply the names of the proteins that were identified in the experimental proteomic datasets (red—protein upregulation, green—protein downregulation) and the color intensities are related to the magnitude of the expression state. This figure was adapted from ref. [8].

Diabetic cardiomyopathy is a form of cardiovascular disease that can afflict people suffering from diabetes mellitus. It is characterized by structural abnormalities and cardiac dysfunction. It can lead to HF and usually the symptoms that otherwise mark HF are not present. Recently, the mitochondrial protein changes associated with diabetic cardiomyopathy have been studied in mice with type 2 diabetes using proteomic profiling [8]. The left heart ventricle tissues of six-month-old male C57BL/6J-lepr/lepr (db/db; diabetic mice) and C57BL/6J (WT) mice were lysed and the proteins were digested using trypsin. The α-amines and ε-amines of the resulting tryptic peptides were labeled with heavy (^13^CD_2_O) or light (CH_2_O) formaldehyde to distinguish between peptides originated from diabetic mice or WT mice. LC-MS/MS (Easy-nLC 1200 coupled to Orbitrap Fusion Lumos Tribrid MS (Thermo Scientific)) was used to analyze the samples. In addition to proteomic analysis, a quantitative evaluation was performed by transthoracic echocardiography and Doppler tissue imaging. They revealed that diabetic mice exhibited diastolic dysfunction and cardiac hypertrophy compared to non-diabetic mice, which confirmed the onset of diabetic cardiomyopathy. A proteomic comparative analysis identified 53 proteins differentially expressed in diabetic mice compared to WT mice, with 30 proteins down-regulated and 23 proteins up-regulated. Among them, the mitochondrial proteins that regulate mitochondrial ATP production and electron transfer in ETC were detected, for example, alpha, beta, delta and epsilon subunits of ATP synthase (ATP5) and cytochrome c1 (complex III) were up-regulated, whilst NDUFB11 and kinase COQ8A (ADCK3) were down-regulated in diabetic mice compared to WT. In order to identify the interconnectivities between proteins (protein–protein interactions) as well as protein biological pathways, the proteins were submitted to the STRING and IPA databases. A STRING analysis of differentially expressed proteins showed enrichment of proteins involved in calcium ion binding (six proteins) and in the striated muscle contraction (two proteins) in WT mice. On the contrary, in diabetic mice, the major enrichment was for the proteins associated with metabolism (nine proteins), FAO (seven proteins) and ATP synthesis (four proteins), with two proteins involved in striated muscle contraction as well. An analysis by IPA indicated OXPHOS and mitochondrial dysfunction as the highest scoring networks in diabetic mice. In addition, differentially expressed proteins were engaged in biological processes such as mitochondrial dysfunction, cardiac fibrosis, cardiac hypertrophy and cardiac necrosis/cell death. Figure 4 shows the IPA analysis of differentially expressed proteins in diabetic mice compared to WT mice with predicted biological processes. To support proteomic data and to investigate the alteration in mitochondrial bioenergetics, a mitochondrial coupling assay between OXPHOS and ETC was performed in mitochondria isolated from left ventricle tissue for both diabetic mice and WT mice. The experiments showed a decreased mitochondrial coupling as well as a reduction in the maximal respiratory capacity in diabetic mice, implying impaired mitochondrial complex I-driven respiration and mitochondria dysfunction. In summary, up-regulation of ATP synthase subunits and cytochrome c1 in the diabetic myocardium supported its attempts for adaptive mechanisms, including the increase in electron transfer and the production of ATP. On the other hand, down-regulation of NDUFB11 and COQ8A, crucial factors that are related to the transfer of electrons to ubiquinone and ubiquinone synthesis, may cause mitochondrial ATP production impairment. The study implied the relation between cardiac dysfunction and the changes in mitochondrial metabolism profile during diabetic cardiomyopathy [8].

Another work that has studied diabetic cardiomyopathy examined the role of SIRT3 in this disease [145]. SIRT3 resides in the mitochondrial matrix and it has been documented that overexpression of SIRT3 in cell cultures promoted respiration and reduced ROS production [146]. In [145], eight-week-old male C57BL/6 mice were injected with streptozotocin (STZ) in order to induce diabetes mellitus, and the protein changes between diabetic and WT mice were analyzed by LC-MS/MS. Twelve weeks after STZ-induced diabetes, the SIRT3 levels decreased in diabetic mice compared to WT and these proteomic data were further validated by real-time PCR and by Western blotting. Furthermore, to examine SIRT3′s role in diabetic cardiomyopathy, SIRT3-KO mice and WT mice injected with STZ were analyzed. SIRT3 deficiency in diabetic SIRT3-KO mice caused increased cardiac dysfunction and myocardial injury, a decrease in ATP levels, an increase in lactate dehydrogenase serum levels and increased ROS accumulation in the myocardium compared to WT diabetic mice. In summary, SIRT3 deficiency worsened diabetic cardiomyopathy in the mice.

## 6. Existing Gaps and Future Directions in Proteomic Research of Mitochondrial Proteome

To understand the causes and mechanisms of disease and determine the appropriate treatments, pathological models are often created. During modeling of pathological conditions related to mitochondrial dysfunction, various drugs (streptozotocin) and metabolic inhibitors (amobarbital, rotenone, etc.) are used. Although use of these pathological models highly contributes to disease characterization, the drugs/inhibitors that are used can exhibit additional effects that can interfere with interpretation of the protein expression and activation status. In addition, these models do not fully reflect the true disease situation which can be affected by variables such as human age, exposure to therapeutic agents and environment. These facts can complicate the results and prevent scientists from coming to the correct conclusions.

Furthermore, mitochondrial proteins are responsible for many cellular functions, and as protein interactions are essential for cellular activities, the proteins do not function as single units but they act as multiprotein complexes. Thus, many pathological alterations can occur because of the disturbances in protein interactions and are not necessary because of protein changes. Hence, the methods that can quantify protein–protein interactions, in addition to the methods that identify the interacting protein partners, are important. In addition, mitochondrial proteins possess structural and biochemical differences and their changes in pathologies should be taken into consideration in addition to the data acquired by proteomics. Therefore, proteomics accompanied by other methods (biochemical, physiological, etc.) can be more informative and can contribute to a more complete mitochondrial protein profiling. Notably, the expression status of mitochondrial proteins identified by MS-based proteomics should be always confirmed and validated by other methods.

The advances in MS-based proteomics minimizes the obstacles related to risk of high false positive rates and identification of low abundance proteins. It is possible to identify and quantify thousands of proteins simultaneously over a high dynamic range and identify mitochondrial proteins from the whole cell proteome. It can be expected in the future that MS-based proteomics will be more often applied to characterization of mitochondrial proteins. Thus, mitochondrial pathways leading to both disease and protection will be described at the protein level. For example, using metabolic labeling of the mitochondrial proteins by non-canonical amino acid AHA could monitor the protein dynamics and allow for identification of newly synthesized proteins in vitro and in vivo in a short time window of a few hours after their synthesis. This could reveal the dysregulation of mitochondrial proteins at early stages of their synthesis and thus earlier recognition of the factors involved in their pathology can be possible as well as the indication of therapeutic targets.

The integrated approach of combining omics technologies in mitochondrial research is a very promising future direction. For example, proteomics magnified and complemented by metabolomic results is especially fitting for investigation of mitochondrial dysfunction and metabolic changes in cardiovascular pathologies. It can show how different expression of proteins affects cellular processes. Several studies have been reported using this approach, and it can be expected that more studies will take advantages of these methods in the future.

However, the major task is to transfer the knowledge obtained in the laboratory to clinical practice. This process is rather slow, although the proteins identified by proteomics as the markers for disease prevention, diagnosis and therapy have found their places in clinics for routine diagnostics. The major hurdles related to this transfer are the quality of sample collection, the complexity of biological sample preparation, instrumentation availability for protein quantity measurements and the accessibility to large patient cohorts for new biomarker validation. 

## 7. Conclusions

The progress in the research of mitochondrial dysfunction-related disorders together with the characterization of the involved mechanisms on a molecular level by highly sensitive instruments in MS-based proteomics has resulted in novel discoveries. These findings have contributed to the explanation of mitochondrial protein interactions and pathways and have indicated protein players associated with pathological conditions. By using MS instruments and developing innovative statistical methods for efficient and accurate analysis of data, investigations of the mitochondrial human proteome are possible, since a small amount of tissue from, e.g., a biopsy, can be sufficient to obtain the relevant data. However, since the mitochondrial proteome is highly dynamic and it changes in both quality and quantity in response to various physiological changes, the important task is to select the right time window and sample type which best represent these changes.

## Figures and Tables

**Table 1:** Examples of the articles which include datasets of mitochondrial proteins and mitochondrial protein databases.

Proteome and/or Database	Description	Year	Ref.
Heart mitochondrial proteome	Protein database comprising 615 proteins identified from purified mitochondria of normal human heart tissue; 1-DE was utilized with protein in-gel digestion and MS identification.	2003	[66]
Mitochondrial proteomes from muscle, liver and heart	MS-based quantitative proteomics revealed striking differences in protein abundances between tissues; new protein residences were confirmed in mitochondria using protein correlation profiling.	2006	[64]
Mitochondrial proteomes from liver, heart, brain and kidney	Mitochondrial proteomes were compared from rat liver, heart, brain and kidney tissues using MS analysis; out of total 8045 proteins, 382 were confirmed to be mitochondrial proteins. Quantitative differences in mitochondrial proteomes of different tissues were detected. A total of 145 mitochondrial proteins were newly identified.	2007	[65]
Mitochondrial complex I	A list of proteins that constitute complex I was generated from different articles on mammals and fungi, in which biochemical methods or deletion of subunit genes were used for identification with subsequent MS analysis; it includes subunit composition, structural data and function.	2016	[52]
FAO pathway	Twenty human proteins (enzymes and transporters) were included in addition to their specific roles in FAO, encoding genes and related disease phenotypes.	2016	[48]
MitoP2	A database that integrates mitochondrial proteins, their molecular functions and associated diseases for yeast, humans and mice.	2006	[69]
MitoCarta3.0	Updated and manually revised genes encoding mammalian mitochondrial proteome from previous MitoCarta and MitoCarta2.0 databases. A total of 1136 human genes and 1140 mouse genes were included that encode proteins, with added annotations of sub-mitochondrial compartments and 149 MitoPathways with MitoPathways Hierarchy. https://www.broadinstitute.org/files/shared/metabolism/mitocarta/mouse.mitocarta3.0.html (accessed on 10 January 2023) https://www.broadinstitute.org/files/shared/metabolism/mitocarta/human.mitocarta3.0.html (accessed on 10 January 2023) https://www.broadinstitute.org/files/shared/metabolism/mitocarta/human.mitocarta3.0.path_.html (accessed on 10 January 2023)	2021	[68]
MitoProteome	The original database that includes 847 human mitochondrial protein sequences obtained from public databases and an MS analysis of purified human mitochondria. The database is updated frequently with data relevant to each protein cross-linked to external databases. http://www.mitoproteome.org (accessed on 10 January 2023)	2004	[70]
MitoMiner v4.0	Originally developed for proteomics, with annotations attached to protein entries, the database was remodeled in 2018 to be gene-centric instead of protein-centric and updated with information on mitochondrial localizations, phenotypes and diseases. http://mitominer.mrc-mbu.cam.ac.uk/ (accessed on 10 January 2023)	2009 2019	[71][72]
MitoCoP	The human mitochondrial high-confidence proteome dataset (>1100 proteins). The resource for placing dynamics, functions, and dysfunctions of mitochondria into the cellular context.	2021	[73]

**Table 2:** The studies of PTMs (acylation and phosphorylation) in various cardiovascular diseases and in other heart conditions that were discussed in detail in Section 4.

PTM	Cardiovascular Disease/Heart Condition	Ref.
Acetylation Malonylation	SLC25A3 deletion-induced mitochondrial cardiomyopathy	[13]
Acetylation	Transverse aortic constriction model of cardiac pressure overload	[11]
Acetylation	Doxorubicin-induced dilated cardiomyopathy	[14]
Acetylation Phosphorylation	Lipid overload-induced cardiomyocyte death, heart hypertrophy and heart dysfunction	[15]
Phosphorylation	Hypertrophic cardiomyopathy	[12]
Phosphorylation	Postnatal development of the heart	[110]
Phosphorylation	I/R and H/R injury with ischemic preconditioning	[111]
Phosphorylation	Ischemia and NOC-18 pretreated ischemic hearts	[46]

## Data Availability

Not applicable.
